# Transcranial direct current stimulation of the prefrontal and visual cortices diversely affects early and late perceptual learning

**DOI:** 10.1002/brb3.3620

**Published:** 2024-07-11

**Authors:** Di Wu, Yan Zhu, Yifan Wang, Na Liu, Pan Zhang

**Affiliations:** ^1^ Department of Medical Psychology Air Force Medical University Xi'an Shaanxi China; ^2^ Department of Neurobiology Basic Medical School Air Force Medical University Xi'an Shaanxi China; ^3^ Department of Nursing Air Force Medical University Xi'an Shaanxi China; ^4^ Department of Psychology Hebei Normal University Shijiazhuang Hebei China

**Keywords:** dorsolateral prefrontal cortex, early and late perceptual learning, middle temporal area, multitarget tDCS, transcranial direct current stimulation (tDCS)

## Abstract

**Background:**

Research has shown that visual perceptual learning (VPL) is related to modifying neural activity in higher level decision‐making regions. However, the causal roles of the prefrontal and visual cortexes in VPL are still unclear. Here, we investigated how anodal transcranial direct current stimulation (tDCS) of the prefrontal and visual cortices modulates VPL in the early and later phases and the role of multiple brain regions.

**Methods:**

Perceptual learning on the coherent motion direction identification task included early and later stages. After early training, participants needed to continuously train to reach a plateau; once the plateau was reached, participants entered a later stage. Sixty participants were randomly divided into five groups. Regardless of the training at the early and later stages, four groups received multitarget tDCS over the right dorsolateral prefrontal cortex (rDLPFC) and right middle temporal area (rMT), single‐target tDCS over the rDLPFC, and single‐target tDCS over the rMT or sham stimulation, and one group was stimulated at the ipsilateral brain region (i.e., left MT).

**Results:**

Compared with sham stimulation, multitarget and two single‐target tDCS over the rDLPFC or rMT improved posttest performance and accelerated learning during the early period. However, multitarget tDCS and two single‐target tDCS led to equivalent benefits for VPL. Additionally, these beneficial effects were absent when anodal tDCS was applied to the ipsilateral brain region. For the later period, the above facilitating effects on VPL induced by multitarget or single‐target tDCS disappeared.

**Conclusions:**

This study suggested the causal role of the prefrontal and visual cortices in visual motion perceptual learning by anodal tDCS but failed to find greater beneficial effects by simultaneously stimulating the prefrontal and visual cortices. Future research should investigate the functional associations between multiple brain regions to further promote VPL.

## Graphical abstract

Multitarget and two single‐target tDCS over the prefrontal and visual cortices improved early learning. Multitarget tDCS and two single‐target tDCS led to equivalent benefits for learning. Neither multitarget nor single‐target tDCS facilitated learning during the later learning.

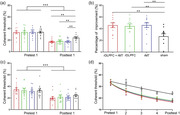

## 1 INTRODUCTION

An intensive behavioral practice that results in large and long‐lasting improvements in visual functions is known as visual perceptual learning (VPL). VPL demonstrates that the human brain is capable of experience‐dependent neural plasticity, particularly in adults who have undergone a critical period of perceptual development during childhood (Bavelier et al., 2010; Kawato et al., 2014). Over the past few decades, the main concerns in the field of VPL have been the locations and forms of plastic changes that occur in the cortex (He et al., 2022). Notably, VPL is gradually making its way into clinics and businesses. There has been significant research showing that VPL is an effective method to enhance or remedy visual functions in both visually impaired populations and healthy people, with large potential applications (Deveau et al., 2013; Lu et al., 2016).

Recently, it has become increasingly popular to couple noninvasive brain stimulation (NIBS) with VPL to enhance visual perception (He et al., 2022; Jia et al., 2022; Liu et al., 2023). On the one hand, NIBS permits the safe modulation of neural processes in particular brain areas, facilitating direct investigation of the causal connection between brain regions and training (Polanía et al., 2018). On the other hand, to achieve satisfactory improvement in visual function, it is often necessary to undergo an extensive period of training (Herpich et al., 2019), which limits the application of VPL to some extent. Fortunately, research has demonstrated that NIBS not only directly improves visual perception (Battaglini et al., 2017; Reinhart et al., 2016) but also enhances VPL when combined with behavioral training (He et al., 2021; Wu et al., 2022).

As an NIBS, transcranial direct current stimulation (tDCS) stands out for its affordability and portability (Reinhart et al., 2016). tDCS temporarily alters cortical excitability by changing the membrane potential of neurons (Stagg & Nitsche, 2011; Stagg et al., 2011). Typically, anodal tDCS enhances cortical excitability, while cathodal tDCS reduces it (Nitsche & Paulus, 2000; Parkin et al., 2015). In a seminal study, stimulating the middle temporal area (MT) or primary motor cortex (M1) with anodal tDCS significantly enhanced the initial learning stage, while cathodal tDCS did not have a notable effect (Antal, Nitsche, Kincses, et al., 2004). Subsequently, many studies have confirmed the effectiveness of tDCS in promoting VPL in both clinical patients (Plow et al., 2012; Spiegel et al., 2013) and healthy people (Sczesny‐Kaiser et al., 2016; Yang et al., 2022).

To our knowledge, all studies regarding VPL coupled with tDCS have used the visual cortex as a stimulation site. However, much evidence suggests that brain plasticity may occur in higher brain regions (e.g., prefrontal or parietal lobes). For example, electrophysiology and brain imaging studies have shown that higher brain regions above the visual cortex, such as the lateral intraparietal area (LIP), the intraparietal sulcus (IPS; associated with the attentional network), and the anterior cingulate cortex (ACC) in the medial frontal cortex, are also involved in VPL (Law & Gold, 2008; Mukai et al., 2007). Additionally, higher cognitive functions (e.g., attention, reward, and decision making) have complex regulatory mechanisms for VPL (Gutnisky et al., 2009; Zhang et al., 2018). These studies provide strong evidence that VPL can also lead to changes in brain areas associated with greater cognitive function. Thus, tDCS contributes to exploring the causality of the prefrontal cortex in VPL.

Previous research has established a foundation for comprehending the advantageous effects of tDCS on VPL. However, these studies were carried out within predetermined and limited training sessions, so they effectively investigated the effects of tDCS only during early training. An unresolved inquiry remains as to whether tDCS continues to enhance later learning effects when performance plateaus. The enhanced learning outcomes in the later plateau phase could have significant and crucial implications for real‐world use since a patient's visual performance level after rehabilitation is directly determined by the level of saturated learning effects. In our previous research, although anodal tDCS over the MT enhanced early visual motion learning, we failed to observe a substantial advantage of anodal tDCS on later learning effects (Wu et al., 2023).

One explanation could be that the visual brain region that was stimulated had a greater impact on visual motion perceptual learning during the initial phase than during the later phase. Numerous studies have indicated that brain regions can undergo dynamic changes throughout the duration of training. After perceptual training, for example, V3A replaces the role of MT in processing noisy motion (Chen et al., 2016), which offers significant proof of the multistage mechanisms of VPL (Shibata et al., 2014; Watanabe & Sasaki, 2015). Thus, the question is how different brain regions play different roles during multistage training.

In conclusion, this study aimed to investigate the causal roles of multiple brain areas (i.e., the frontal cortex and visual cortex) in early and later visual motion learning using anodal tDCS. Previous studies have suggested that both the left MT (lMT) and right MT (rMT) may be effective stimulation targets for improving motion perception (Battaglini et al., 2017; Wu et al., 2020; Zito et al., 2015). The presentation of a stimulus in the contralateral visual field may generate a greater stimulation effect if only one hemisphere is targeted by the stimulation. Many previous studies have also presented stimuli in the contralateral visual field (He et al., 2022; Herpich et al., 2019). In the present study, the contralateral hemisphere was stimulated under experimental conditions, and the ipsilateral hemisphere was stimulated under control conditions. Specifically, anodal tDCS was applied to the right dorsolateral prefrontal cortex (rDLPFC) and the rMT, the rDLPFC alone, and the rMT alone, while participants trained on a coherent motion direction identification task. As a control location, the lMT was also stimulated since the target stimuli were displayed on the left visual field. The entire training process included the early and later stages. The former included the first three training sessions, and the latter included three continuous training sessions after the performance plateaued. We hypothesized that (a) multitarget tDCS (rDLPFC + rMT) would have a greater beneficial effect on early and later visual motion learning than single‐target tDCS and that (b) both single‐target tDCS on the rDLPFC and rMT, relative to lMT and sham stimulation, could improve VPL.

## 2 MATERIALS AND METHODS

### 2.1 Participants

Sixty college students (mean age = 20.30 ± 1.42 years, range = 18–24 years, 14 females) were randomly assigned to five groups: (1) multitarget anodal tDCS on the rDLPFC + rMT, *n* = 12; (2) anodal tDCS on the rDLPFC, *n* = 12; (3) anodal tDCS on the rMT, *n* = 12; (4) anodal tDCS on the lMT, *n* = 12; and (5) sham stimulation, *n* = 12. Table 1 shows the matched demographic characteristics among the five groups. All participants were right‐handed, with normal neurology and normal or corrected‐to‐normal vision. Informed consent was obtained in advance. The study was approved by the Research Ethics Committee and performed in accordance with the ethical principles of the Declaration of Helsinki.

**TABLE 1:** Demographic characteristics (numbers or means and standard deviations).

Variable	rDLPFC + rMT	rDLPFC	rMT	lMT	sham	*F*/*χ* ^2^	*p*
Gender (males)	9	10	9	8	10	1.30^a^	0.861
Age (years)	20.25 (1.71)	20.33 (1.30)	20.58 (1.44)	20.25 (1.28)	20.08 (1.50)	0.19^b^	0.944
Vision (logMAR)	−0.12 (0.04)	−0.13 (0.05)	−0.12 (0.05)	−0.13 (0.04)	−0.13 (0.04)	0.12^b^	0.976
*n*	12	12	12	12	12		

Abbreviations: lMT, left middle temporal; rDLPFC, right dorsolateral prefrontal cortex; rMT, right middle temporal.

^a^ *χ* ^2^ test.

^b^ one‐way analysis of variance.

### 2.2 Apparatus

The experiment was performed in a quiet, dark room. At a distance of 57 cm, participants binocularly observed the display, which covered an area of 6.84° × 3.89°. Stabilization of the participants’ heads was achieved by combining a chinrest with a forehead bar. A gamma‐corrected monitor (1920 × 1080 pixel spatial resolution; 60 × 34 cm size; 85 Hz refresh rate) was used to display visual stimuli, with a computer running PsychToolbox extensions in MATLAB. The stimuli were displayed in the visual periphery, so an eye tracking system (EyeLink 1000 Plus) was used to monitor eye fixation in real time by measuring the pupil center position and corneal reflection with an infrared camera. When the eye exceeded a 1.5° distance from the fixation point in any direction during stimulus presentation, the experiment stopped, and continuous tones sounded. The trial was restarted once the eye returned to the range of fixation.

### 2.3 Design and procedure

**FIGURE 1:**
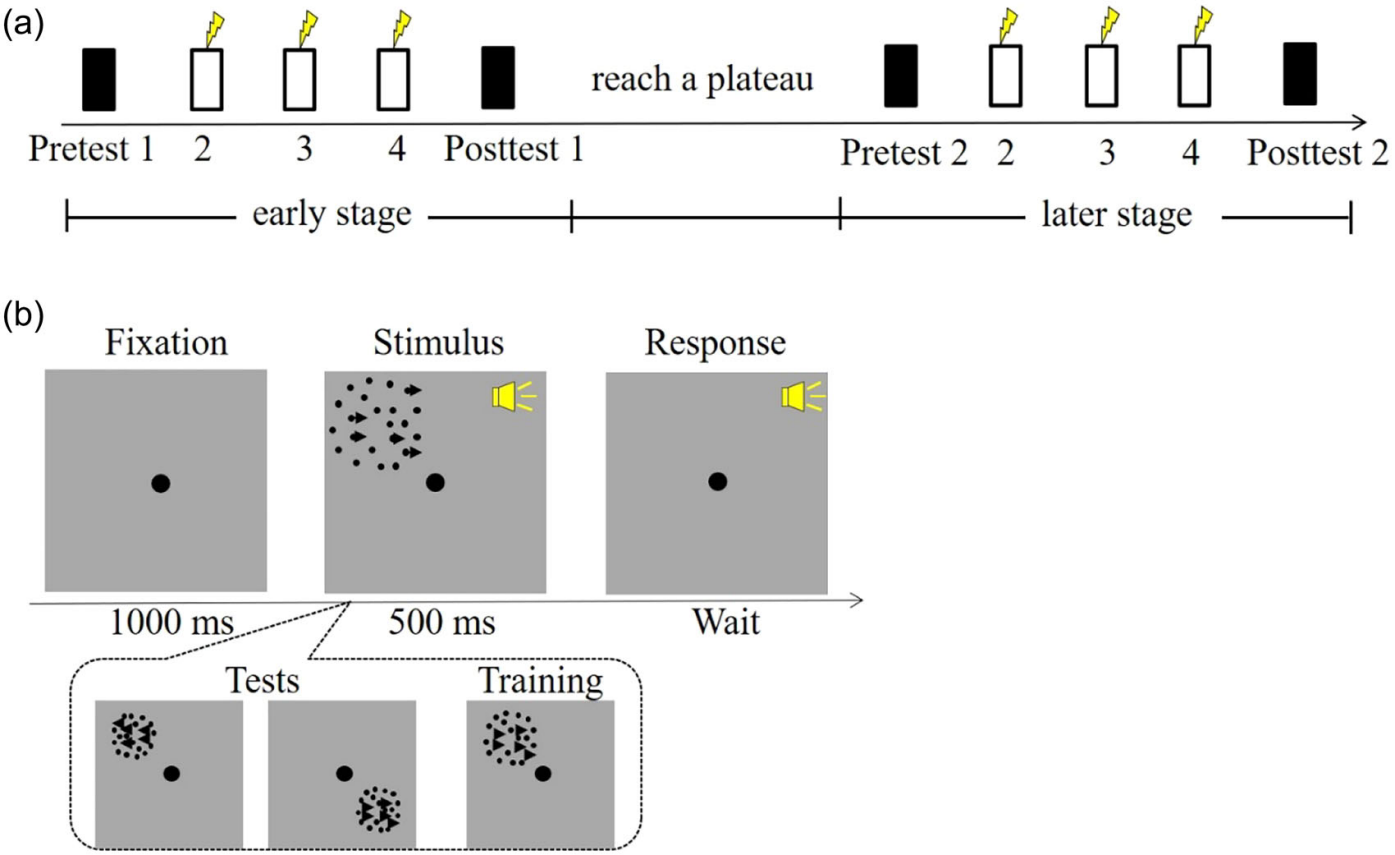
Procedure and task. (a) The yellow lightning bolts indicate that anodal (or sham) transcranial direct current stimulation (tDCS) was administered. The black and white rectangles represent the test and training sessions, respectively. (b) An example of a coherent motion direction identification trial.

The experimental process had two stages: early and late (Figure 1a). The early stage included early training sessions, including pretest 1, 3‐day training with stimulation, and posttest 1. At the completion of the early stage, all participants continued to train until the performance plateaued. The later stage represented the later training sessions, including pretest 2, 3 days of training with stimulation, and posttest 2. Two points are worth mentioning. (1) No stimulation was delivered in the intermediate period between the early and later stages. (2) The definition of the learning plateau in this study was a coherent threshold reduction of <1% on 2 consecutive days, and the average number of continued training sessions during the intermediate period was 2.85 ± 1.35 sessions.

### 2.4 Experimental task

A 1000‐ms fixation dot (0.4° in diameter) was presented on a blank screen at the beginning of a trial, immediately followed by the 500‐ms stimulus accompanied by a brief tone. Upon the stimulus disappearing, participants pressed the left or right arrow keys to judge the global direction of coherently moving dots in a two‐alternative forced choice (2AFC) task. The stimulus consisted of 51 black moving dots (diameter: 0.06°, speed: 10°/s, and contrast: 100%), which were presented on a gray background with 26 cd/m^2^ luminance. Moving dots were randomly presented within a 5°‐diameter round window in the first frame. New dots replaced the ones that moved outside the window at random, new locations within the window, thereby keeping the moving dot density constant (2.6 dots/deg^2^). A subset of dots (coherently moving dots) was moved in one of the left or right directions; other dots were moved randomly (Figure 1b).

During the tests, each response was followed by a brief tone regardless of accuracy. Participants were asked to separately complete two types of tests in which the stimulus was in the visual periphery centered either at the top left (−5°, 5°) or at the bottom right (5°, −5°) relative to the fixation point. It took approximately 5 min to finish one test with 120 trials. During the training, only correct responses were accompanied by a brief tone. It took approximately 15.3 ± 0.75 min to complete one training session that consisted of five blocks of 70 trials. The time of each training session was shorter than the 20‐min stimulation period to ensure simultaneous stimulation with training. Participants had a self‐timed break after the completion of each block.

The percentage of coherently moving dots converged to 79.4% correct through an adaptive three‐down/one‐up staircase method. The coherent threshold decreased by 10% when there were three consecutive correct responses; in contrast, the coherent threshold increased by 10% once one response was incorrect. A reversal occurred once the direction of the staircase changed, that is, a decreasing threshold switched from an increasing threshold or vice versa. After removing the first four (if the reversals were even) or five (if odd) reversals, the averaged remaining reversals were the coherent threshold.

### 2.5 Stimulation protocols

**FIGURE 2:**
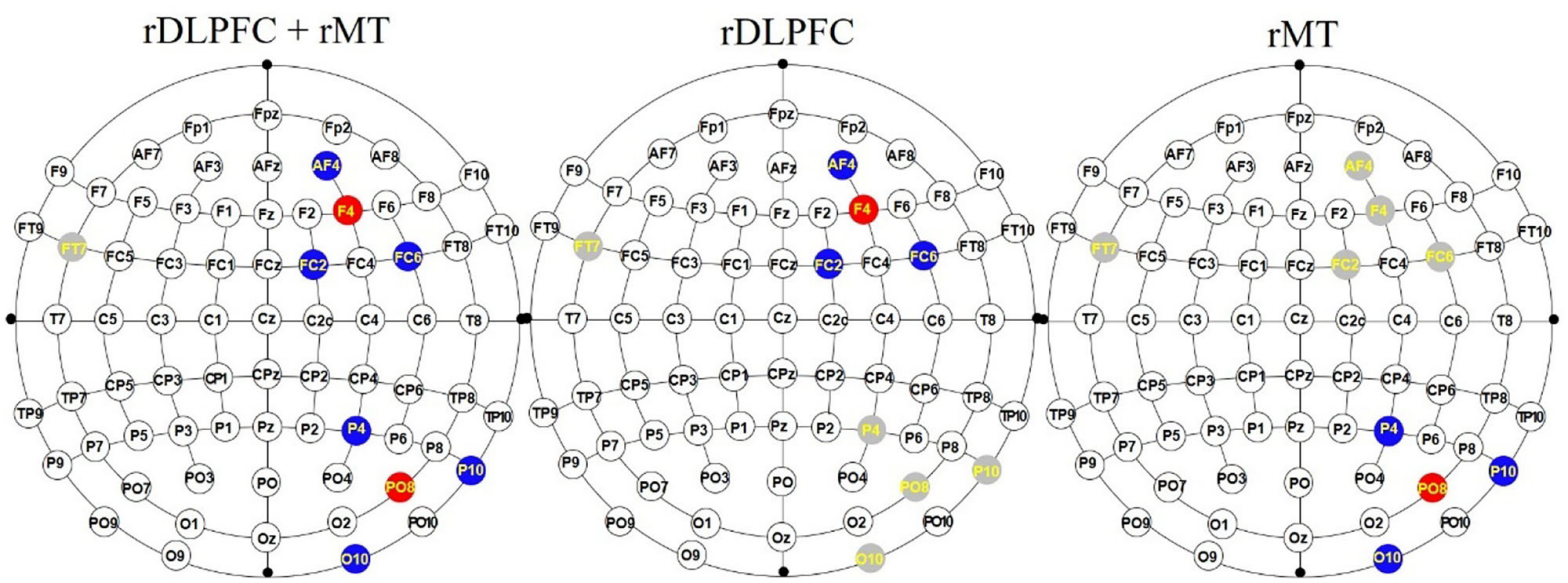
Location of each electrode for each stimulation group. The red circles represent the central electrodes, and the current intensity is 1.5 mA for each electrode. The blue circles are the return electrodes, and the current intensity is −0.5 mA for each electrode. The gray circles are the electrodes with a current intensity of 0 mA.

High‐definition tDCS (HD‐tDCS) was administered by a nine‐channel stimulator (Soterix Medical Inc.) since the advantage of this approach is that the current approach can be focused more directly on the target brain regions using small electrodes than the conventional method using large sponge electrodes (Dmochowski et al., 2011). Two wires from the stimulator were used to stimulate two brain regions. As shown in Figure 2, the first wire, which included five thin wires with electrodes, delivered direct current to the rDLPFC; three return electrodes (AF4, FC2, and FC6; 10‐10 standard electroencephalogram system) surrounded the central electrode at F4. The remaining electrode was placed on an FT7 for grounding. The second wire was used to deliver direct current to the rMT through diverging four wires with electrodes; the central electrode was positioned at PO8, along with three return electrodes at P4, P10, and O10.

**FIGURE 3:**
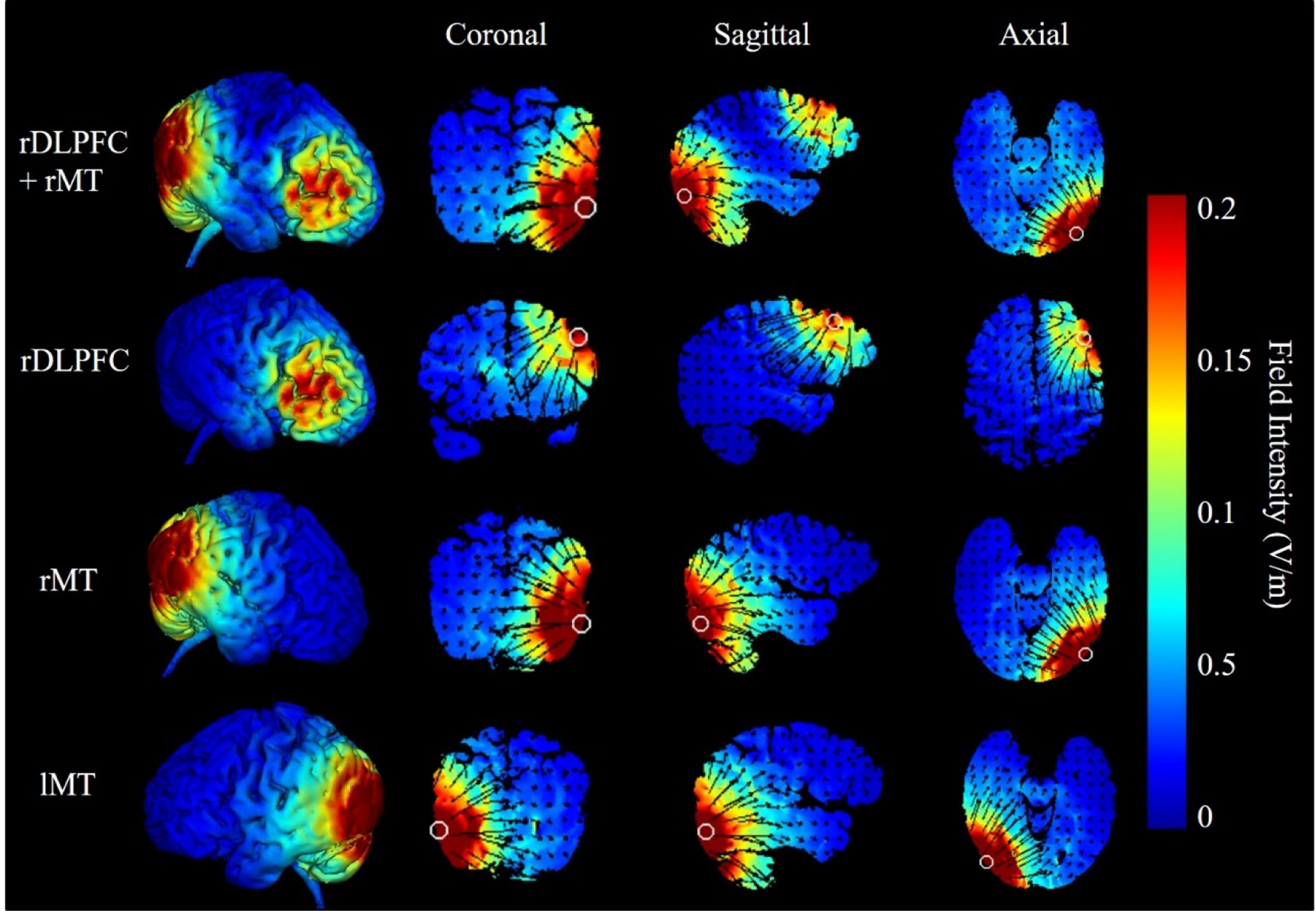
Field intensity and current flow were simulated using HD‐Explore software.

For the rDLPFC + rMT group, two central electrodes (F4 and PO8) separately delivered a 1.5 mA direct current to the remaining electrodes. The rDLPFC and rMT groups had the same electrode positions as the multitarget group (rDLPFC + rMT), and only the currents were altered to achieve blinding. Specifically, the direct current delivered from the second wire was set to 0 mA for the rDLPFC condition, and the direct current delivered from the first wire was set to 0 mA for the rMT condition. For the lMT condition, the positions of all electrodes were symmetric to those of the rMT condition. Sham‐stimulated participants received rDLPFC + rMT, rDLPFC, or rMT stimulation. During the 20 min period, the direct current was ramped up for 30 s and ramped down for 30 s. The impedance of each electrode was kept below 5 kΩ through the conductive gel in electrode casings. HD‐Explore software was used to simulate the current flow, as shown in Figure 3.

During each training session, the following question was asked orally, and participants reported their tDCS‐induced sensations: “How are you feeling in the two stimulated areas, including pain, itchiness, burning, tingling, and so on? A 10‐point scale was used to record sensation intensity: 0 = none to 10 = strong and intolerable. Please report your sensations separately.” After the early and later stages, participants were asked to report twice whether they received real or sham stimulation during the three training sessions.

### 2.6 Data analysis

The mean percentage improvement, which compared performance in the posttraining measurement to the pretraining baseline, was calculated as follows:

$$\begin{array}{ccc} & & \mathrm{Percent}\;\mathrm{improvement}\;\\ & & =\frac{\mathrm{threshold}\;\mathrm{in}\;\mathrm{pretest}-\mathrm{threshold}\;\mathrm{in}\;\mathrm{posttest}}{\mathrm{threhold}\;\mathrm{in}\;\mathrm{pretest}}\;\times 100\% \end{array}$$

The learning curves, illustrating the threshold dynamics over the training time, were fitted by a power function during early and later training sessions. The power function:

$$C\left(t\right)=\;C_{0}\;\times \;t^{-\rho }$$

where $C_{0}$ is the initial threshold, t is the session number, and ρ is the learning rate.

Using nonlinear least squares, we minimized the sum of the squared differences between the predicted and measured values.

The goodness of fit was estimated by

$$r^{2}=1.0-\frac{\sum {\left(y_{\mathrm{measured}}-y_{\mathrm{predicted}}\right)}^{2}}{\sum {\left[y_{\mathrm{measured}}-\mathrm{mean}\left(y_{\mathrm{predicted}}\right)\right]}^{2}}$$

where $y_{\mathrm{measured}}\;$ and $y_{\mathrm{predicted}}$ represent the measured and predicted values, respectively. $\mathrm{mean}(y_{\mathrm{measured}})$ is the mean of all the measured values.

An *F* test was used to statistically compare the nested models:

$$F\left(df_{1},df_{2}\right)\;=\;\frac{\left(r_{\mathrm{full}}^{2}-r_{\mathrm{reduced}}^{2}\right)/df_{1}}{\left(1-r_{full}^{2}\right)/df_{2}}$$

where $df_{1}=k_{full}\;-k_{\mathrm{reduced}}$, $df_{2}=\;N-k_{full}$, $k_{full}$ and $k_{\mathrm{reduced}}$ are the numbers of parameters of the full and reduced models, respectively, and *N* is the number of data points.

In addition to the frequentist statistical approaches, Bayesian analyses were performed with the opensource software package JASP. Bayesian analyses permit a test of the relative strength of evidence for the null hypothesis (H0: no effect of tDCS stimulation) versus the alternative hypothesis (H1: change in behavior as a result of tDCS stimulation). Two‐way Bayesian analysis of variance on coherence thresholds were performed in JASP.

## 3 RESULTS

### 3.1 Anodal tDCS modulated visual motion learning during early training sessions

**FIGURE 4:**
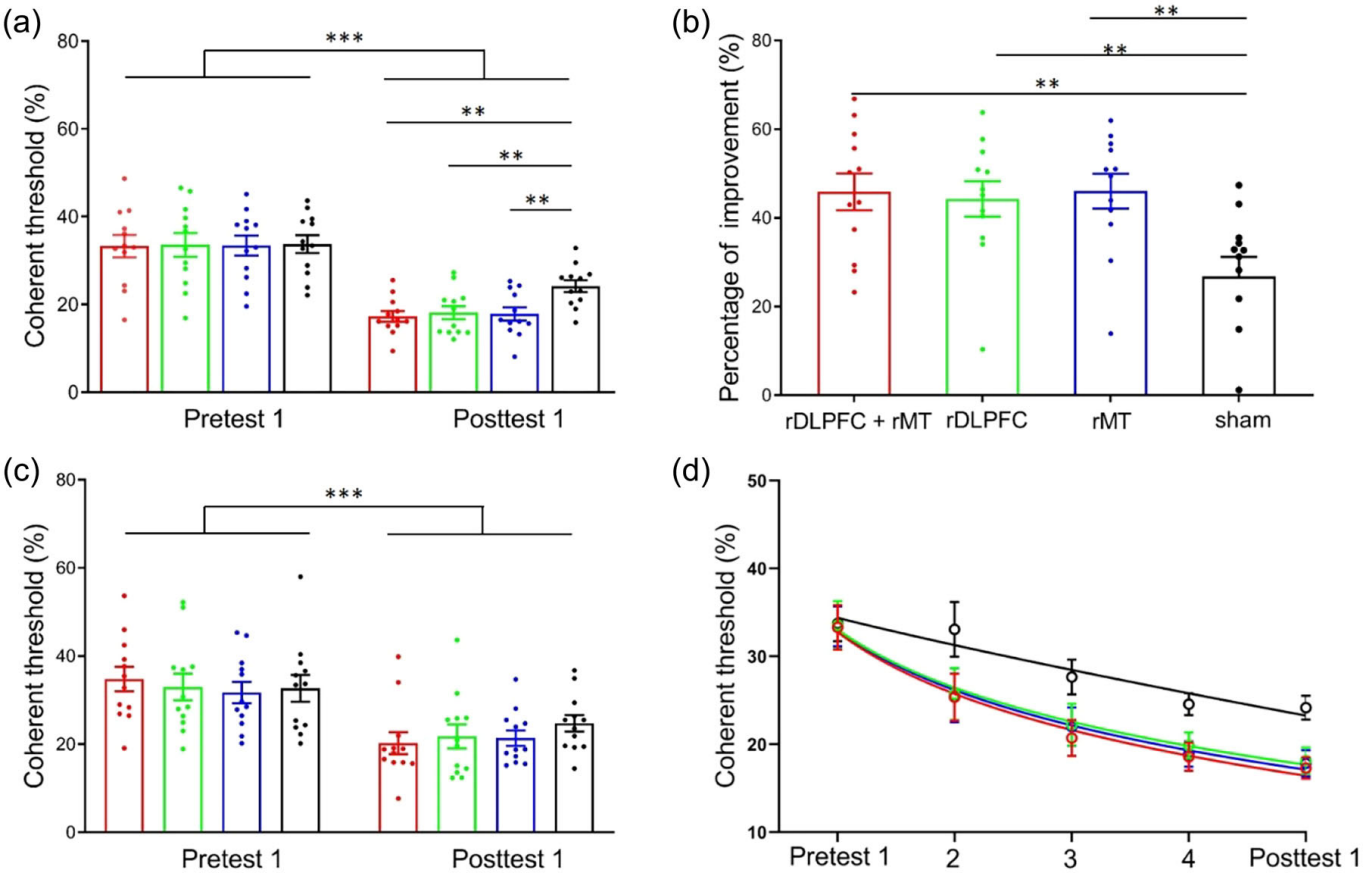
Anodal transcranial direct current stimulation (tDCS) effects during the early training sessions. (a) The coherence threshold measured by pre‐ and posttest 1 when stimuli were presented at the trained position (top left). (b) Percent improvement. (c) Learning generalization: the coherent threshold measured by pre‐ and posttest 1 when stimuli were displayed at an untrained position (bottom right). (d) Learning curves: a power function‐fitted learning curve represents the average threshold of participants at two tests and three training sessions. For all subgraphs, red, green, blue, and black represent the rDLPFC + rMT, right dorsolateral prefrontal cortex (rDLPFC), right middle temporal (rMT), and sham stimulation groups, respectively. The data are presented as the means (bars) ± standard error of means (error bars).

First, two‐way analysis of variance and Bayesian analysis of variance were performed on the coherent thresholds for stimuli at the trained position (i.e., top left) with the two tests (pretest 1 and posttest 1) as within‐subject factors and the four groups (rDLPFC + rMT, rDLPFC, rMT, and sham) as between‐subject factors (Figure 4a). The main effect of *test* was significant, with *F*(1,44) = 204.03, *p* < .001, *η* ^2 ^= .80, and *BF*_10_ = 7.95E+17. Additionally, the interaction effect reached marginal significance, *F*(3,44) = 2.37, *p* = .083, and *η* ^2 ^= .03. The Bayes factor for the *test* × *group* interaction effect (alternative hypothesis H1: significant interaction effect) was between 1 and 3 (BF10 = 1.03), providing anecdotal evidence supporting H1. There was no significant main effect of *group*, with *F* < 1 and *BF*_10_ = 0.33. A consistent pattern of results was found across both frequentist and Bayesian analyses. We aimed to investigate the threshold differences among the four groups. Notably, the post hoc least significant difference (LSD) test did not show a significant difference in the thresholds of pretest 1 among the four groups, *F* < 1, indicating successful random grouping; in contrast, the posttest 1 thresholds of the four groups were significantly different from each other, *F*(3,44) = 5.28, *p* = .003, and *η* ^2 ^= .26. Furthermore, the posttest 1 thresholds for all three real stimulation groups (the rDLPFC + rMT, the rDLPFC, and the rMT) were significantly lower than those for the sham group, with *p* values of .001, .004, and .003, respectively. There were no significant differences between the three real stimulation groups (*p* > .1). These results suggested that although three real stimulations facilitated VPL compared with sham stimulation, multitarget tDCS did not promote training compared with single‐target tDCS.

Second, we calculated the percentage of coherent threshold improvements between pre‐ and posttest 1 (Figure 4b). One‐way analysis of variance revealed that the percent improvement significantly differed among the four groups (*F*(3,44) = 5.15, *p* = .004, and *η* ^2 ^= .13). Further analyses revealed a greater percentage of improvement in the three real stimulation groups (the rDLPFC + rMT, the rDLPFC, and the rMT) than in the sham group (*p* = .002, .004, and .002, respectively). Similarly, the percentage improvement between the three groups of real stimulation did not differ significantly (*p* > .1).

Third, we investigated the effects of anodal tDCS on the generalizability of VPL findings. Thus, two‐way analysis of variance of the coherence threshold at the untrained position (bottom right) was conducted (Figure 4c). The main effects of the *test* on the thresholds were significant, with *F*(1,44) = 72.44, *p* < .001, *η* ^2 ^= .60, and *BF*_10_ = 9.54E+8. The main *group* was not significant, *F*(1,44) = 1.12, *p* = .351, *η* ^2 ^= .03, and *BF*_10_ = 0.17, and interaction effects were also not significant, with *F* < 1, *BF*_10_ = 0.32. Furthermore, an LSD test showed a nonsignificant difference in the threshold of the untrained position among the four stimulation groups regardless of pretest 1 or posttest 2 (all *p*s > .1). It appears that tDCS did not affect the generalizability of VPL during early training sessions.

Figure 4d shows the coherent threshold learning curves for the four stimulation groups during the early training session, fitted with a power function. The ninth model (M9) was statistically equal to the full model and superior to another reduced model, which provided the best‐fitting model (Figure S1 and Table S1). M9 had identical initial thresholds ($C_{0}$) for all four learning curves and identical learning rates (ρ) for the three real stimulation (rDLPFC + rMT, rDLPFC, and rMT) learning curves, and there was a significant difference in learning rates (ρ) between the three active stimulation and sham groups. This best‐fitting model demonstrated that all three stimulation protocols accelerated the learning process related to sham stimulation, but there were no differences in learning rate among the three methods.

### 3.2 Anodal tDCS failed to influence learning in the later training period

We analyzed the coherence threshold when performance plateaued. The data were analyzed in the same way as the data from the earlier training. First, the coherence threshold measured at the trained position (top left) between pretest 2 and posttest 2 was analyzed by two‐way analysis of variance. A significant main effect of *test* was found, *F*(1,44) = 6.36, *p* = .015, *η* ^2 ^= .12, and *BF*_10_ = 3.77. However, no other significant effects regarding *group* or interaction were observed, *F*s < 1, *BF*_10(group) _= .35, *BF*_10(interaction) _= .14.

Next, we compared the percent improvement between the four groups by one‐way analysis of variance. The results did not show a significant difference in the percentage of improvement, *F *< 1. These results demonstrated that relative to sham stimulation, neither multitarget nor single‐target tDCS enabled participants to achieve greater improvement in the trained position when plateau performance was reached.

We also investigated generalization in later training sessions. Two‐way analysis of variance on the threshold measured at the untrained position (bottom right) was conducted. The main effect of *test* was significant, *F*(1,44) = 4.54, *p* = .039, *η* ^2 ^= .09, *BF*_10_ = 1.59. Additionally, neither the main effect of *group* nor the interaction effect were significant (*F*s < 1, *BF*_10(group) _= 0.235, and *BF*_10(interaction) _= 0.16), suggesting that various stimulation methods did not influence generalization when the plateau was reached.

**FIGURE 5:**
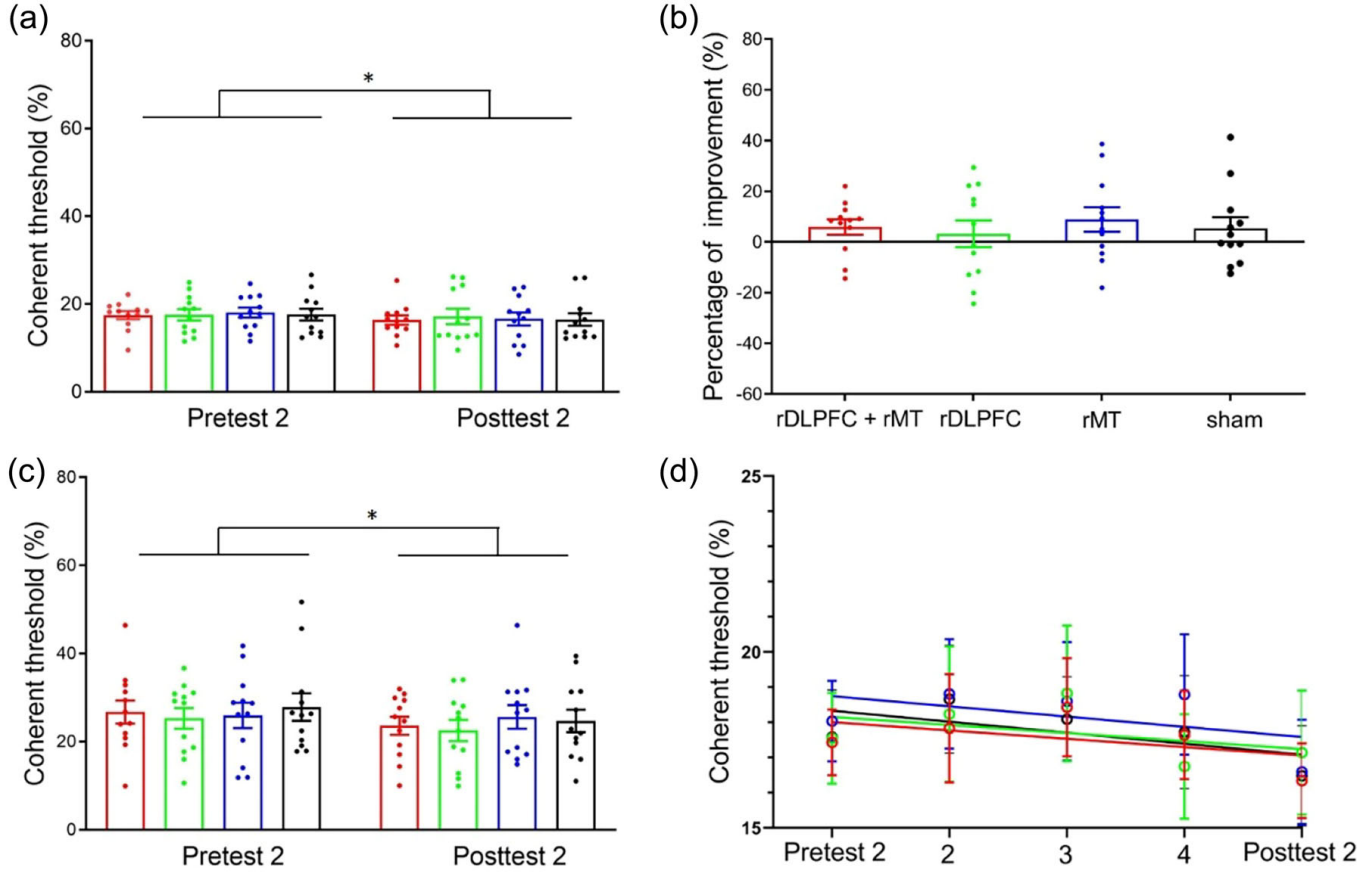
Anodal transcranial direct current stimulation (tDCS) effects during the later training sessions. (a) The threshold measured by pre‐ and posttest 2 when stimuli were presented at the trained position (top left). (b) Percent improvement. (c) Learning generalization: the threshold measured by pre‐ and posttest 2 when stimuli were displayed at an untrained position (bottom right). (d) Learning curves. For all subgraphs, red, green, blue, and black represent the rDLPFC + rMT, right dorsolateral prefrontal cortex (rDLPFC), right middle temporal (rMT), and sham stimulation groups, respectively.

Finally, we fitted the four learning curves during the later training session (Figure 5d). The model comparison results showed statistically similar results for the full and reduced models. In other words, the four learning curves had identical initial thresholds ($C_{0}$) and learning rates (ρ) when the performance plateaued. These results indicated that any stimulation montages were not able to affect the learning process once the performance was saturated.

### 3.3 Anodal tDCS modulated learning in a location‐specific manner

**FIGURE 6:**
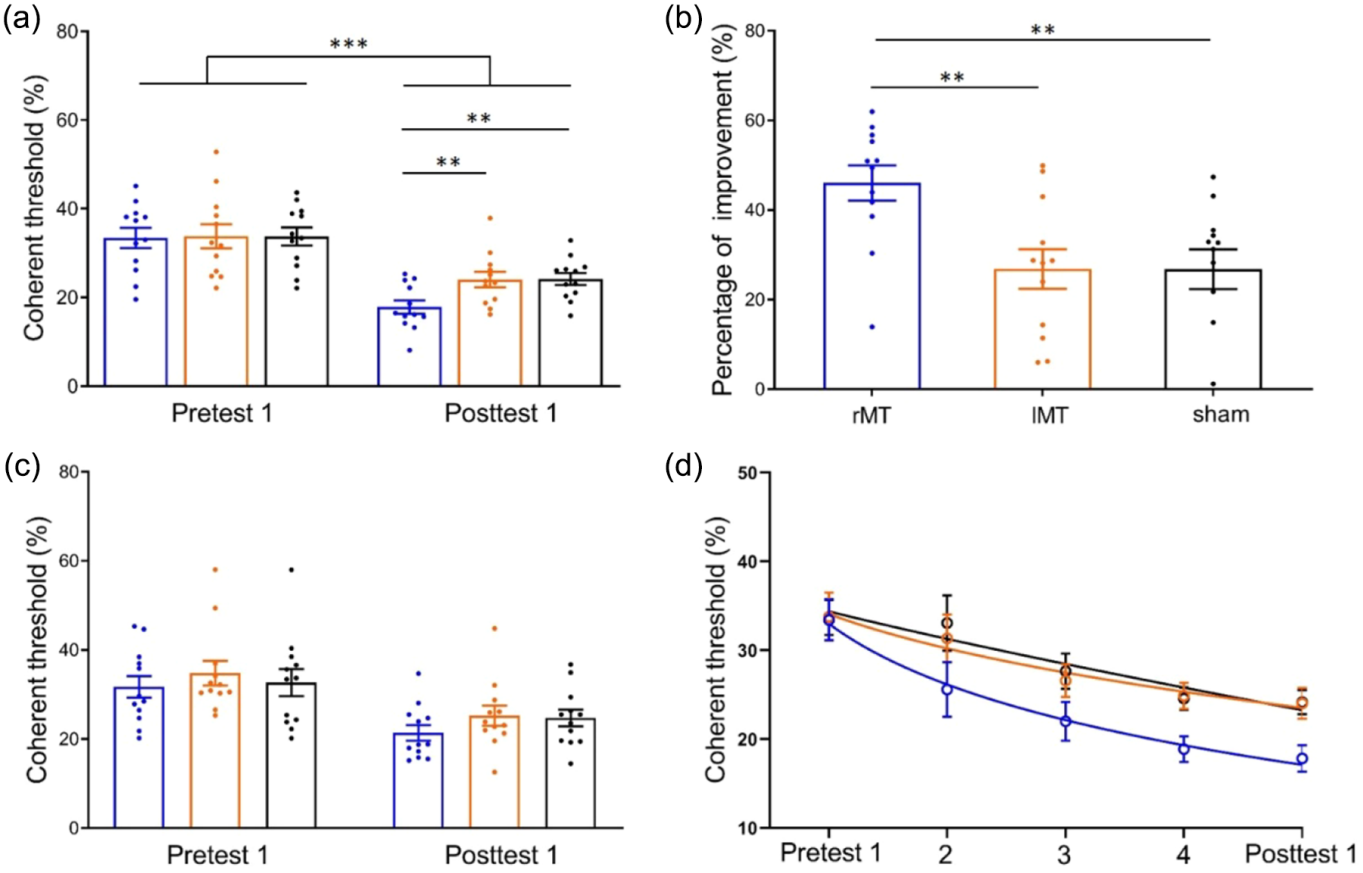
Results of the control experiment in which anodal transcranial direct current stimulation (tDCS) was administered to the ipsilateral brain region. (a) Coherent thresholds of the trained position (top left) at pretest 1 and posttest 1. (b) Percent improvement. (c) Learning generalization: coherent thresholds of the untrained position (bottom right) at pretest 1 and posttest 1. (d) Learning curves. For all subgraphs, blue, orange, and black represent the right middle temporal (rMT), left middle temporal (lMT), and sham stimulation groups, respectively.

The above results showed a beneficial effect of three real stimulation protocols on coherent motion direction identification. One question was whether this beneficial effect comes from the participants’ cutaneous sensation caused by tDCS. To exclude the possibility of placebo effects on tDCS modulatory effects, one additional group of participants (lMT) was trained on coherent motion identification, while their left MT region was stimulated by anodal tDCS. Two‐way analysis of variance was performed on the coherent thresholds of the trained location with three *groups* (rMT, lMT, and sham groups) as a between‐subject factor and two *tests* (pretest 1 and posttest 1) as a within‐subjects factor (Figure 6a). A significant main effect of *test* was found, *F*(1,33) = 113.16, *p* < .001, *η* ^2 ^= .74, and *BF*_10_ = 1.64E+10. Additionally, the interaction effect was marginally significant, *F*(2,33) = 3.25, *p* = .052, *η* ^2 ^= .04, and *BF*_10_ = 1.50, providing anecdotal evidence supporting H1 (alternative hypothesis H1: significant interaction effect). However, the *group* main effect was not significant, *F*(2,33) = 1.18, *p* = .319, *η* ^2 ^= .07, and *BF*_10_ = 0.504. The frequentist and Bayesian analyses revealed a consistent pattern of results. For the three groups of participants, their thresholds of training location at pretest 1 were the same, *F* < 1; in contrast, the thresholds at posttest 1 were significantly different, *F*(2,33) = 5.50, *p* = .009, and *η* ^2 ^= .25. The LSD showed a lower threshold in the rMT group than in the lMT (*p* = .008) and sham groups (*p* = .007). However, there was no significant difference in the threshold between the lMT and sham groups (*p* = .956).

Next, we also found significant differences in percent improvement among the three groups, *F*(2,33) = 6.80, *p* = .003, and *η* ^2 ^= .29. The LSD test showed a greater percentage improvement in the rMT group than in the lMT group (*p* = .003) and in the sham group (*p* = .003), while no significant difference between the lMT group and the sham group was found (*p* = .993) (Figure 6b).

Additionally, we investigated the generalization of VPL among the three groups (Figure 6c). Two‐way analysis of variance on the thresholds at untrained locations showed a significant main effect of *test*, *F*(1,33) = 33.49, *p* < .001, *η* ^2 ^= .50, and *BF*_10_ = 7.04E+4, and no other significant effects were found, *Fs* < 1, *BF*_10(group) _= 0.33, and *BF*_10(interaction) _= 0.21.

Finally, the three learning curves were fitted by a power function (Figure 6d). The fourth model (M4) was regarded as the best‐fitting model (Figure S2 and Table S2) since it was statistically equal to the full model and superior to the other reduced models. M4 had identical initial thresholds ($C_{0}$) for all three learning curves but a significant difference in learning rates (ρ) between the rMT and lMT/sham groups. This best‐fitting model demonstrated that contralateral stimulation can boost the learning process related to ipsilateral and sham stimulation.

In short, the above results indicated that ipsilateral stimulation (i.e., lMT) did not facilitate visual motion learning compared with contralateral stimulation (i.e., rMT), supporting the location‐specific manner in which anodal tDCS modulates learning, which contributed to excluding a possible placebo effect.

### 3.4 Subjective cutaneous sensation

**FIGURE 7:**
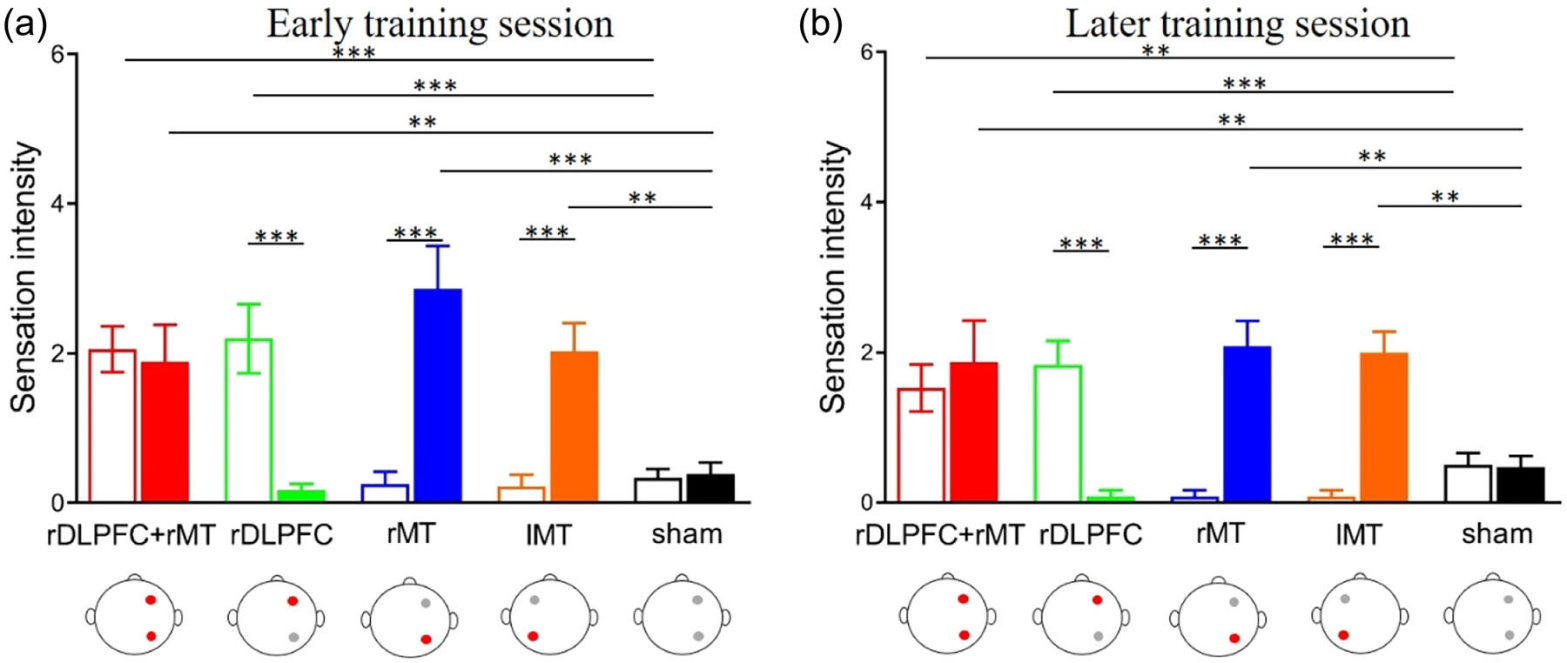
Sensation intensity in the prefrontal and visual cortexes. (a) Sensation intensity during early training sessions. (b) Sensation intensity during later training sessions. Red, green, blue, orange, and black represent the rDLPFC + rMT, right dorsolateral prefrontal cortex (rDLPFC), right middle temporal (rMT), left middle temporal (lMT), and sham stimulation groups, respectively. The hollow columns represent the sensation intensity in the prefrontal cortex, and the solid columns show the sensation intensity in the visual cortex. The red dots in the head model indicate the real stimulated brain regions and the gray dots represent the sham‐stimulated brain regions.

We compared the cutaneous sensation of two brain areas among the five groups, which contributed to providing evidence for the effectiveness of our stimulation protocols. We calculated the average sensation intensity by averaging the sensation intensity across three training sessions. Thus, the average sensation intensity at early or later training periods was separately calculated. Two‐way analysis of variances with five *groups* (rDLPFC + rMT, rDLPFC, rMT, lMT, and sham) as a between‐subject factor and two *locations* (prefrontal cortex and visual cortex) as a within‐subjects factor were conducted for the averaged sensation intensity at early or later training periods. For the early training session (Figure 7a), the main effect of *location* was significant, *F*(1,55) = 7.10, *p* = .010, *η* ^2 ^= .05, and *BF*_10_ = 0.97; additionally, a significant main effect of *group* was also observed, *F*(4,55) = 4.78, *p* = .002, *η* ^2 ^= .26, and *BF*_10_ = 4.33. Importantly, the interaction effect was also significant, *F*(4,55) = 22.55, *p* < .001, *η* ^2 ^= .59 and *BF*_10_ = 0.48E+10. Furthermore, we analyzed the difference in sensation intensity between the rDLPFC and the rMT/lMT in each group. The *p‐*values for the five groups were .665, <.001, <.001, <.001, and .885, respectively. Additionally, participants in the rDLPFC + rMT and rDLPFC groups felt stronger sensation in the rDLPFC area than did those in the rMT, lMT, and sham groups, *p*s < .001; correspondingly, participants in the rDLPFC + rMT, rMT, and lMT groups had stronger sensation in the rMT/lMT area than did those in the rDLPFC and sham groups, *p*s < .001.

For the later training session (Figure 7b), main effects of *location* and *group* were found, *F*(1,55) = 13.52, *p* = .08, *η* ^2 ^= .05, and *BF*_10(location)_ = 3.02; *F*(4,55) = 3.55, *p* = .012, *η* ^2 ^= .26, and *BF*_10(group)_ = 1.39. A significant interaction effect was also found, *F*(4,55) = 26.31, *p* < .001, *η* ^2 ^= .20, and *BF*_10_ = 5.40E+10. Furthermore, the differences in sensation intensity between the rDLPFC and the rMT/lMT in each group were analyzed, and the *p‐*values for the five groups were .256, <.001, <.001, <.001, and .927. Additionally, the rDLPFC + rMT and rDLPFC groups exhibited stronger sensations in the rDLPFC than did the rMT, lMT, and sham groups (*p* < .001), and the rDLPFC + rMT, rMT, and lMT groups exhibited stronger sensations in the rMT/lMT area than did the rDLPFC and sham groups (*p* < .001). Similar results were found between the early and later training sessions. The above results indicated a more obvious sensation in the brain area that received active stimulation, helping us judge the effectiveness of the stimulation.

Finally, we analyzed the subjective expectations of the real and sham groups. For the early training sessions, the *χ* ^2^ test did not show a significant difference in subjective expectations among the five groups, *χ* ^2 ^= 6.01, *p *= .198. For the later training session, no significant difference in subjective expectations was found, *χ* ^2 ^= 6.30, *p *= .178. Generally, tDCS‐induced sensations were perceived to be stronger than sham‐induced sensations, and real tDCS cannot be distinguished from sham tDCS.

## 4 DISCUSSION

We found that anodal tDCS over the prefrontal and visual cortexes had dissociable effects on early and later learning. In the early stage, multitarget tDCS (rDLPFC + rMT) or single‐target tDCS (rDLPFC or rMT), relative to sham stimulation, resulted in greater improvement and accelerated learning. However, stimulating two brain regions generated comparable performance compared to stimulating a single brain region. Additionally, beneficial effects were absent when the ipsilateral brain region (lMT) was stimulated by anodal tDCS. In the later stage, neither multitarget nor single‐target tDCS facilitated coherent motion identification learning. Using a noninvasive neuromodulation method, we demonstrated the involvement of the rDLPFC in visual motion perception learning, enhancing the understanding of the causal role of the prefrontal cortex in VPL. In addition, this is the first study to examine the effect of multiple brain region stimulations on perceptual training, contributing to the development of a new simulation method for clinical applications.

Our study demonstrated that anodal tDCS over the rDLPFC effectively enhanced visual motion perceptual learning. Several studies have demonstrated that the prefrontal cortex plays a pivotal role in learning a visual task (Jia et al., 2018; Kahnt et al., 2011) since it is responsible for both decision making and memory (Euston et al., 2012). For example, a neuroimaging study showed that the neural activity of the mPFC increased following visual motion perpetual learning and was correlated with the amount of learning across participants (Larcombe et al., 2018). An electrophysiological study also revealed that animals’ behavioral choices were entirely related to a component of the neural code that emerged earlier in the prefrontal cortex and not in the V4 cortex, and its enhancement completely generalized to an untrained stimulus configuration (Jing et al., 2021). However, these studies are able to reveal the correlations between the brain and behavior, failing to investigate causal brain–behavior relationships. To fill this gap, the current study confirmed the causal role of the prefrontal cortex in VPL with anodal tDCS.

Consistent with our previous findings, this study further confirmed the causal role of MT in motion perceptual learning by tDCS. Indeed, a variety of techniques have confirmed MT involvement in visual motion perception, such as electrophysiology (Britten et al., 1992), lesion imaging (Newsome & Pare, 1988), brain imaging (Chen et al., 2016), and stimulation (Antal, Nitsche, Kruse, et al., 2004). Previously, we showed that applying anodal tDCS over the MT for 20 min improved motion perception, suggesting that the MT is involved in visual motion perception (Wu et al., 2020).

We found that multitarget tDCS was not more conducive to improving visual motion perceptual learning than single‐target tDCS, which was not in line with our expectation. In other areas of research, for example, tDCS simultaneously stimulating the right inferior frontal gyrus and the presupplementary motor area improves response inhibition more than stimulating a single brain region (Guo et al., 2022). In addition, gait freezing was reduced when the M1 and left dorsolateral prefrontal cortex (lDLPFC) were stimulated simultaneously but not when the M1 was stimulated or when the lDLPFC was stimulated alone (Dagan et al., 2018). Based on previous research, visual motion perceptual learning is related to the prefrontal and visual cortexes, and it is reasonable to speculate that simultaneously exciting two brain regions by anodal tDCS produces greater beneficial effects on learning than exciting a single brain region. However, our results did not support this hypothesis. The reason may be that two brain regions may have complex and refined connectivity that is not captured by mere simultaneous stimulation and that more training benefits could be gained through more refined stimulation. The “reweighting hypothesis” suggests that perceptual learning incrementally optimizes the connections between sensory representations and decisions (Dosher & Lu, 1998; Dosher et al., 2013). Thus, we suggest that functional coupling between the prefrontal cortex and visual cortex should be investigated first in future research and that functional coupling be further modulated by transcranial alternating current stimulation (tACS), which contributes to revealing the functional connection between two brain regions during perceptual learning.

Interestingly, the motion direction identification task is categorized within mid‐level VPL, which uses patterns composed of simple visual features (Lu & Dosher, 2022). Correspondingly, VPL in high‐level tasks (e.g., object identification, face perception, and biological motion) involve objects and natural scenes and are processed by high‐level cortical areas, such as the inferior temporal cortex and prefrontal cortex. Relative to mid‐level tasks, VPL in high‐level tasks often might reflect the creation of new high‐level representations of objects or scenes by combining existing visual features (Lu & Dosher, 2022). Thus, it is reasonable to anticipate a more pronounced effect of frontal tDCS when training with high‐level tasks, which is worthy of further study.

Similar to our previous finding (Wu et al., 2023), only early training showed a tDCS effect; in contrast, this effect gradually declined over time. Specifically, anodal tDCS did not improve learning at the later stage, even though the prefrontal and visual cortexes were simultaneously or solely stimulated. However, our results cannot indicate that tDCS is unable to influence the later learning effect, that is, further performance improvement induced by tDCS after reaching the plateau period. The possible reason may be that we have not yet found an appropriate stimulation approach. For example, the prefrontal and visual cortex could be functionally coupled to produce better stimulation effects. It would be very valuable to answer this question since it would help reveal the associations between the cortical loci where plastic changes occur and develop a new stimulation approach to maximize training effects.

Another possibility for the noninfluence of anodal tDCS on the later learning effect could be attributed to participants reaching a ceiling effect that was challenging to modulate through tDCS stimulation. We used the adaptive three‐down/one‐up staircase method to control the thresholds during training. Although this method is commonly used in current VPL research, it may not precisely gauge the tiny amount of threshold change induced by tDCS when participants’ performances approach the asymptote. Thus, it is possible that the influence of tDCS on learning effects at later stages (plateau level) is very slight, which is difficult to observe through the measurement method adopted in this study.

One limitation of this study was the number of participants. Variability in tDCS effects has resulted in calls for greatly increased sample sizes (Minarik et al., 2016). Our sample size (*n* = 12 per group) was comparable to or greater than several tDCS studies in the VPL that found significant effects (Herpich et al., 2019; Sczesny‐Kaiser et al., 2016), although it was smaller than some studies (; Pirulli et al., 2013). Given the small sample size and the complexity of the study design, future research should increase the credibility of the findings with a larger sample size.

## 5 CONCLUSIONS

In conclusion, compared with ipsilateral stimulation and sham stimulation, multitarget or single‐target tDCS over the contralateral brain region enhanced early visual motion learning. However, multitarget tDCS generated a performance comparable to that of single‐target tDCS. Additionally, neither multitarget nor single‐target tDCS improved learning at later stages, as evidenced by the posttest performance and learning curves during the plateau period. Future studies should explore functional coupling between cortical loci during training and then modulate functional coupling by noninvasive stimulation methods (e.g., tACS) to further explore the role of multiple brain regions in VPL.

## AUTHOR CONTRIBUTIONS

*Conceptualization*: Di Wu and Pan Zhang. *Methodology*: Di Wu and Pan Zhang. *Investigation*: Di Wu, Yan Zhu, and Yifan Wang. *Formal analysis*: Di Wu and Na Liu. *Data curation*: Di Wu, Na Liu, and Yan Zhu. *Resources*: Pan Zhang. *Writing—original draft*: Di Wu and Yan Zhu. *Writing—review and editing*: Di Wu and Pan Zhang. *Visualization*: Di Wu. *Project administration*: Di Wu, Na Liu, and Pan Zhang. *Funding acquisition*: Di Wu, Na Liu, and Pan Zhang.

## CONFLICT OF INTEREST STATEMENT

The authors declare no conflicts of interest.

### PEER REVIEW

The peer review history for this article is available at https://publons.com/publon/10.1002/brb3.3620.

## Supporting information


**FIGURE S1** The learning curves (rDLPFC + rMT, rDLPFC, rMT and sham) of each model were fitted by power functions. Red, green, blue and black represent the rDLPFC + rMT, rDLPFC, rMT and sham stimulation groups, respectively.
**FIGURE S2** The learning curves (rMT, lMT and sham) of each model were fitted by power functions. Blue, orange and black represent the rMT, lMT and sham stimulation groups, respectively.
**TABLE S1** Comparison of model fits to the four learning curves during the early training sessions.
**TABLE S2** Comparison of model fits to the three learning curves during the early training sessions.

## Data Availability

The data are available upon request from the first author.
