# Supplementary material for: Transcranial direct current stimulation of the prefrontal and visual cortices diversely affects early and late perceptual learning
Source: Brain Behav. 2024 Jul 11;14(7):e3620. doi: 10.1002/brb3.3620 (PMC11238241; doi:10.1002/brb3.3620)
Supplement: Supplementary file 1 — FIGURE S1 The learning curves (rDLPFC + rMT, rDLPFC, rMT and sham) of each model were fitted by power functions. Red, green, blue and black represent the rDLPFC + rMT, rDLPFC, rMT and sham stimulation groups, respectively. FIGURE S2 The learning curves (rMT, lMT and sham) of each model were fitted by power functions. Blue, orange and black represent the rMT, lMT and sham stimulation groups, respectively. TABLE S1 Comparison of model fits to the four learning curves during the early training sessions. TABLE S2 Comparison of model fits to the three learning curves during the early training sessions. [file BRB3-14-e3620-s001.docx]

**Supplementary Information**

**Learning curves in the rDLPFC + rMT, rDLPFC, rMT and sham groups**

We estimated the four learning curves (rDLPFC + rMT, rDLPFC, rMT and sham) by power functions with a total of 8 parameters. The learning curve was the coherent threshold as a function of pretest 1, 3 training sessions and posttest 1. All participants in each session were averaged to calculate the value of each session. Each learning curve has two parameters: the initial threshold ($C_{0}$) and learning rates ($\rho$). Since we have confirmed that the averaged coherent thresholds in the four groups were not significantly different, it is possible to hypothesize that the four groups had the same initial threshold $C_{0}$. Thus, twelve models were developed by setting learning rates ($\rho$) equal to one another and simultaneously keeping the initial threshold ($C_{0}$) the same (Table S1). Specifically, the model lattice consisted of 12 models, including the full 5-parameter model (M1) with identical $C_{0}$ and independent $\rho$ values; the reduced 4-parameter model with identical $C_{0}$ and three independent $\rho$ (M2, M3 M4, M5, M6 and M7) values; the reduced 3-parameter model with identical $C_{0}$ and two independent $\rho$ values (M8, M9, M10 and M11); and the reduced 2-parameter model (M12) with identical $C_{0}$ and $\rho$ values.


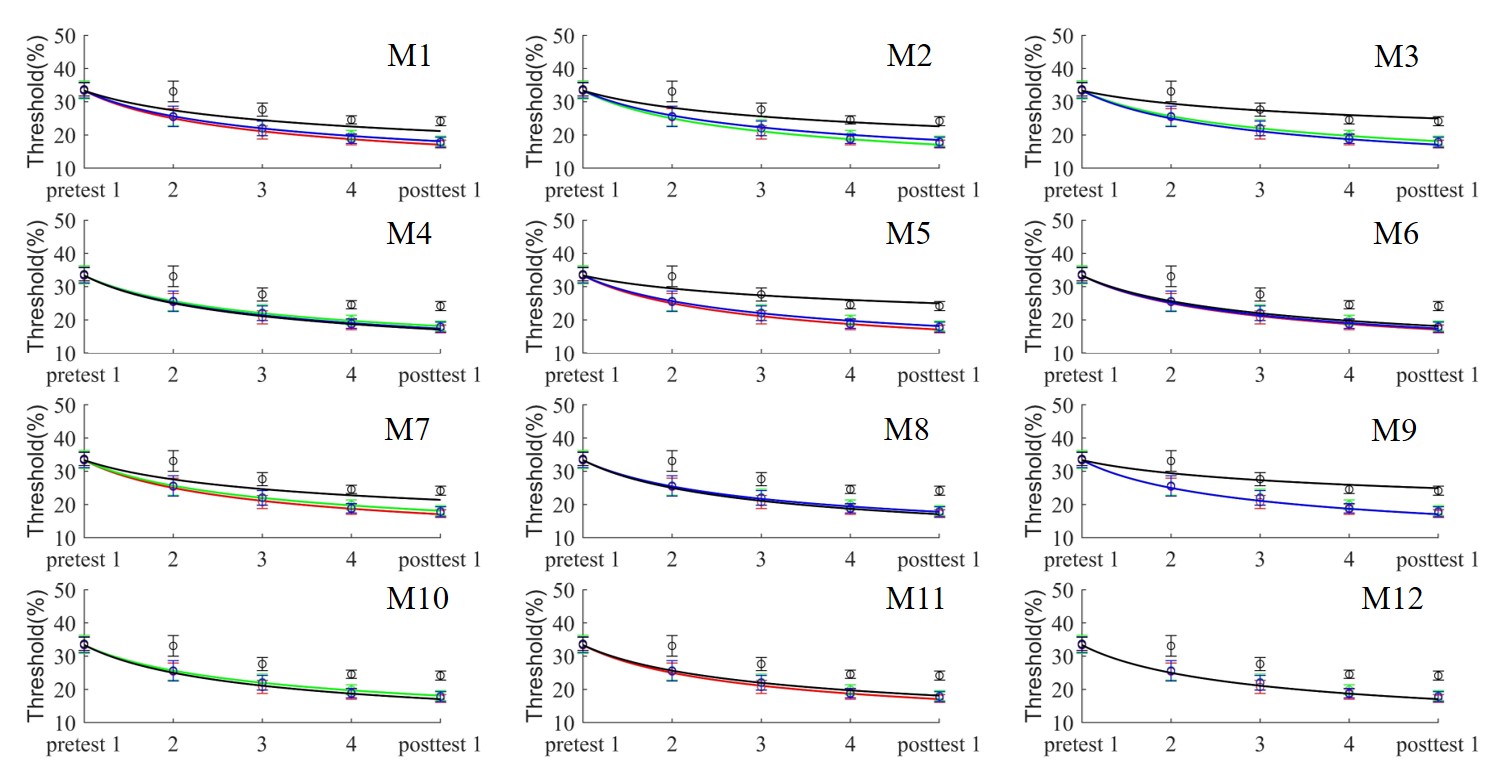


**FIGURE S1** The learning curves (rDLPFC + rMT, rDLPFC, rMT and sham) of each model were fitted by power functions. Red, green, blue and black represent the rDLPFC + rMT, rDLPFC, rMT and sham stimulation groups, respectively.

**Learning curve in the rMT, lMT and sham groups**

Three learning curves (rMT, lMT and sham) were fitted by power functions with a total of 6 parameters. Each learning curve has two parameters: the initial threshold ($C_{0}$) and learning rates ($\rho$). Similarly, we hypothesized the same initial threshold $C_{0}$ for the three groups. Thus, we developed five models by manipulating learning rates ($\rho$) equal to one another and simultaneously kept the initial threshold ($C_{0}$) the same (Table S2). Specifically, the model lattice consisted of 5 models, including the full 4-parameter model (M1) with identical $C_{0}$ and independent $\rho$ values; the reduced 3-parameter model with identical $C_{0}$ and two independent $\rho$ (M2, M3 and M4) values; and the reduced 2-parameter model (M5) with identical $C_{0}$ and $\rho$ values.


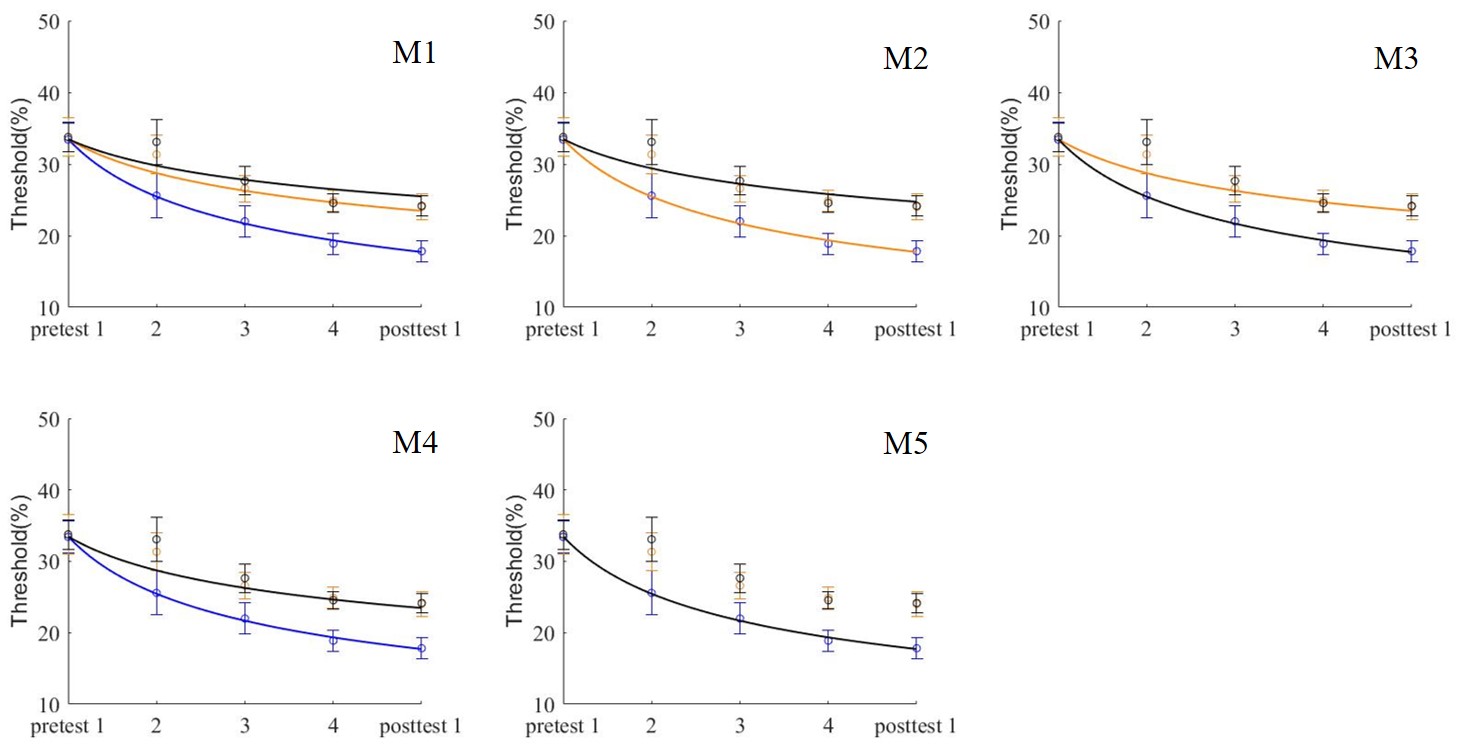


**FIGURE S2** The learning curves (rMT, lMT and sham) of each model were fitted by power functions. Blue, orange and black represent the rMT, lMT and sham stimulation groups, respectively.

**TABLE S1** Comparison of model fits to the four learning curves during the early training sessions.

|  | M2 | M3 | M4 | M5 | M6 | M7 | M8 | M9 | M10 | M11 | M12 | $r^{2}$(%) | *n* | Parameters |
| --- | --- | --- | --- | --- | --- | --- | --- | --- | --- | --- | --- | --- | --- | --- |
| M1 | 0.251 | 0.105 | 1 | 0.101 | 1 | 1 | 1 | 0.306 | 1 | 1 | 1 | 91.46 | 5 | ${1C}_{0},4\rho$ |
| M2 |  |  |  |  |  |  | 1 | 0.306 | 1 | 1 | 1 | 94.30 | 4 | ${1C}_{0}, 3\rho;\rho_{1}=\rho_{2}$ |
| M3 |  |  |  |  |  |  | 1 | 1 | 1 | 1 | 1 | 97.21 | 4 | ${1C}_{0}, 3\rho;\rho_{1}=\rho_{3}$ |
| M4 |  |  |  |  |  |  | 1 | <0.001 | 1 | <0.001 | 1 | 70.65 | 4 | ${1C}_{0}, 3\rho;\rho_{1}=\rho_{4}$ |
| M5 |  |  |  |  |  |  | 1 | 1 | 1 | 1 | 1 | 97.36 | 4 | ${1C}_{0}, 3\rho;\rho_{2}=\rho_{3}$ |
| M6 |  |  |  |  |  |  | 1 | 0.002 | 1 | 1 | 1 | 77.50 | 4 | ${1C}_{0}, 3\rho;\rho_{2}=\rho_{4}$ |
| M7 |  |  |  |  |  |  | 1 | 0.027 | 1 | 1 | 1 | 86.41 | 4 | ${1C}_{0}, 3\rho;\rho_{3}=\rho_{4}$ |
| M8 |  |  |  |  |  |  |  |  |  |  | 1 | 70.09 | 3 | ${1C}_{0}, 2\rho;\rho_{1}=\rho_{2}=\rho_{4}$ |
| M9 |  |  |  |  |  |  |  |  |  |  | 1 | 96.58 | 3 | ${1C}_{0}, 2\rho;\rho_{1}=\rho_{2}=\rho_{3}$ |
| M10 |  |  |  |  |  |  |  |  |  |  | 1 | 70.53 | 3 | ${1C}_{0}, 2\rho;\rho_{1}=\rho_{3}=\rho_{4}$ |
| M11 |  |  |  |  |  |  |  |  |  |  | 1 | 77.50 | 3 | ${1C}_{0}, 2\rho;\rho_{2}=\rho_{3}=\rho_{4}$ |
| M12 |  |  |  |  |  |  |  |  |  |  |  | 69.88 | 2 | ${1C}_{0}, 2\rho$ |

Notes: Columns 2 to 12 display the *p* values of statistical comparisons between pairs of models. *n* represents the number of each model parameter. The model parameters are shown in the right column.

**TABLE S2** Comparison of model fits to the three learning curves during the early training sessions.

|  | M2 | M3 | M4 | M5 | $r^{2}$(%) | *n* | Parameters |
| --- | --- | --- | --- | --- | --- | --- | --- |
| M1 | < 0.001 | < 0.001 | 0.126 | 0 | 94.07 | 4 | ${1C}_{0},3\rho$ |
| M2 |  |  |  | < 0.001 | 63.07 | 3 | ${1C}_{0}, 2\rho;\rho_{1}=\rho_{2}$ |
| M3 |  |  |  | < 0.001 | 56.77 | 3 | ${1C}_{0}, 2\rho;\rho_{1}=\rho_{3}$ |
| M4 |  |  |  | 0 | 93.61 | 3 | ${1C}_{0}, 2\rho;\rho_{2}=\rho_{3}$ |
| M5 |  |  |  |  | 25.77 | 2 | ${1C}_{0}, 1\rho$ |

Notes: Columns 2 to 5 display the *p* values of statistical comparisons between pairs of models. *n* represents the number of each model parameter. The model parameters are shown in the right column.
